# Glass Formation in the GeSe_2_–As_2_Se_3_–MeCh Systems (Me = Cu, Ag, Zn, Cd, Sn, Pb; Ch = S, Se, Te)

**DOI:** 10.3390/ma18215058

**Published:** 2025-11-06

**Authors:** Lilia Aljihmani

**Affiliations:** Faculty of Metallurgy and Materials Science, University of Chemical Technology and Metallurgy, 8 Kliment Ohridski Blvd., 1797 Sofia, Bulgaria; l_aljihmani@uctm.edu

**Keywords:** chalcogenide glasses, glass formation, glass-forming region, synthesis

## Abstract

The creation of novel, effective materials with specific properties is necessary to advance technology. To do this, objective regularities between the material’s composition, structure, and properties must be found. A comparative analysis of glass-forming regions, arranged according to the systematic substitution of one element by its analog within a periodic system subgroup, provides a useful framework for discussing trends in glass formation in semiconductor alloys. In this review, the information on the glass formation in the chalcogenide systems GeSe_2_–As_2_Se_3_–MeCh, where Me = Cu, Ag, Zn, Cd, Sn, Pb; Ch = Se, Te, was subjected to a thorough comparative analysis to establish objective patterns in the change in the glass-forming ability in these systems. The effect of MeCh on the formation of glass in the binary system GeSe_2_–As_2_Se_3_ was traced.

## 1. Introduction

Glassy chalcogenide semiconductors are distinguished by a number of unique properties, with only a small part of these properties being manifested or reported to be absent in crystalline semiconductors, especially their capacity for memory and reversible electrical switching [1,2,3], phenomenal radiation resistance [4,5], photostructural transformations [6,7], absence of the influence of impurities [8], the need to use inherently pure materials in synthesis, simple technology, etc. They find application in fields of technology, including electronics, and have long been implemented in production: black-and-white and color vidicons [9,10], coatings [11], photo- [12], electronic, and X-ray resists [9], electrical switches and memory elements [13], optical details for the infrared region [14], passive and active components and systems for fiber [15] and integrated optics [16], devices for reversible information recording with a capacity of more than billions of bits on a single disk, etc. [12,17,18,19,20].

Chalcogenide glasses have been the subject of extensive research since their discovery in the mid-twentieth century. In the 1950s and 1960s, research focused on synthesizing and characterizing glass formation in basic systems, such as As_2_S_3_ and As_2_Se_3_, which exhibited distinct optical and electrical properties. Subsequent research advanced our understanding of glass formation, structural organization, and the impacts of compositional alteration and dopants. Later, efforts focused on enhancing physical, thermal, and optical properties by improved synthesis control, compositional tweaking, and nanostructuring. More recently, research has focused on the functional applications of chalcogenide glasses in infrared optics, photonics, and phase-change memory systems, aided by computational modeling and sophisticated characterization techniques.

The search for new chalcogenide glasses has been put on a qualitatively new footing in recent years. The previously dominant approach for synthesizing new chalcogenide glasses in two-, three-, and more-element systems has been replaced by a new one: the synthesis of new chalcogenide glasses in multicomponent systems. The essence of this approach is based on the empirical “like dissolves in like” rule. In the previous approach, the starting components are elements (2, 3, and more); most often, one is a chalcogen element, the second is a metal, and the third is a dielectric or semiconductor. In the new approach, the initial components are two (or more) chalcogenides of the type Me_n_Ch_m_ (Me = metal; Ch = S, Se, or Te). This approach ensures a significantly higher metal content in the glass, which is crucial for its use in most cases.

The development of technologies requires new, highly efficient materials with predetermined properties. This necessitates finding objective regularities between the material’s structure, composition, and properties. The consideration of glass formation in specific classes of semiconductor alloys is carried out based on a systematic comparative analysis of glass-forming regions, arranged according to the substitution of one element by its analog within a periodic system subgroup. Such an approach enables tracing periodic features in glass formation, which have significant predictive value for the search for new glassy systems.

Despite extensive research on the characteristics of Ge–As–Se chalcogenide glasses, a comprehensive comparison of systems modified with metallic chalcogenides has not yet been provided. The GeSe_2_–As_2_Se_3_–MeCh (MeCh—metal chalcogenide) systems are selected for this review because they represent one of the most versatile families of chalcogenide glasses. Both GeSe_2_ and As_2_Se_3_ are well-known glass formers capable of producing stable amorphous networks with advantageous thermal and optical characteristics. The incorporation of various metal chalcogenides enables systematic tuning of structural, optical, and electrical properties, making these systems highly relevant for fundamental research and technological applications. Moreover, understanding the effect of metal chalcogenide additions on the glass-forming region provides valuable insight into structure–property relationships in complex chalcogenide materials.

This review provides a comprehensive summary of the available information on the synthesis procedures and glass-forming region of GeSe_2_–As_2_Se_3_-based chalcogenide glasses. Particular emphasis is placed on the influence of metal chalcogenide additions on glass formation and related properties. Although the glass-forming region of a given system, once reported, is seldom re-examined, variations in synthesis conditions can significantly modify this region. Accordingly, the present work aims to consolidate existing data, evaluate the effects of compositional and synthesis parameters, and offer an updated perspective on glass formation behavior in these systems.

## 2. Glass Formation in the GeSe_2_–As_2_Se_3_–MeCh Systems (Me = Cu, Ag, Zn, Cd, Sn, Pb; Ch = Se, Te)

### 2.1. Glass Formation in the GeSe_2_–As_2_Se_3_–Cu_2_Se System [21,22]

The glass formation in the GeSe_2_–As_2_Se_3_–Cu_2_Se system is delineated using 73 samples synthesized by a direct monotemperature method from high-purity elements [21]. After synthesis at 1100 °C, the samples are cooled in water. The samples are analyzed using X-ray diffraction (powder diffractometer DRON 4–13, CuK_α_ radiation).

**Figure 1:**
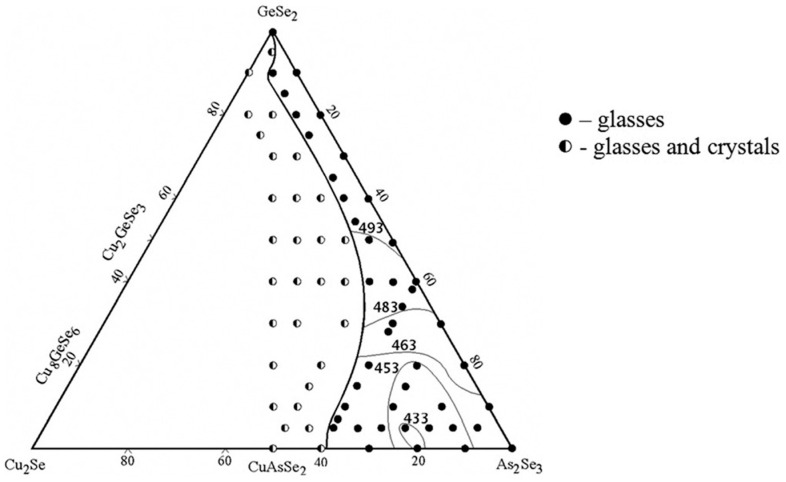
Glass formation in the GeSe_2_–As_2_Se_3_–Cu_2_Se system. Reprinted with permission from Ref. [21]. Copyright 2014 AIP Publishing.

The glassy state is characterized by the lack of peaks on the diffractograms, the presence of an amorphous halo, the uniformity of the polished surface when observed with a metallographic microscope, and the characteristic shape of the differential heating and cooling curves. XRD results confirmed the formation of three ternary compounds, Cu_8_GeSe_6_ (space group P6_3_cm), Cu_2_GeSe_3_ (space group Imm2), and CuAsSe_2_ (space group R3), with the diffraction patterns accurately indexed according to the crystallographic planes [22]. The glass-forming region of the GeSe_2_–As_2_Se_3_–Cu_2_Se system is presented in Figure 1.

It extends along the length of the GeSe_2_–As_2_Se_3_ system, expanding significantly with increasing As_2_Se_3_ content. At As_2_Se_3_ contents of 0–45 mol%, the glass-forming region lies parallel to the GeSe_2_–As_2_Se_3_ side, with a Cu_2_Se content below 10 mol%. In the 60–100 mol% As_2_Se_3_ range, the maximum Cu_2_Se content reaches around 38 mol% [21].

### 2.2. Glass Formation in the GeSe_2_–As_2_Se_3_–Ag_2_Se System [23,24,25]

To delineate the glass-forming area in the GeSe_2_–As_2_Se_3_–Ag_2_Se system, samples (3 g) of elements with high purity are synthesized in evacuated (up to 10^−3^ mm Hg) quartz ampoules and heated to 600 °C and from 600 °C to 980 °C at 5 °C/min and 2 °C/min, respectively [23,24]. The melt is homogenized in a shaking furnace. The holding time at the maximum synthesis temperature is 4 h. After cooling to 850 °C, the melt is quenched in an ice-water bath. The alloys are subjected to stabilizing annealing at a temperature 30 °C below the softening point for 3 h. The glassy state is verified by the absence of crystalline inclusions and phase separation, as observed under a microscope on the polished surfaces of the samples. XRD analysis is conducted to verify the structural state of the samples. The results confirmed the presence of four indexed ternary compounds: orthorhombic Ag_8_GeSe_6_, hexagonal Ag_3_AsSe_3_, tetragonal LTM–AgAsSe_2_, and AgAs_3_Se_5_, indicating the formation of crystalline phases within the studied system [25].

**Figure 2:**
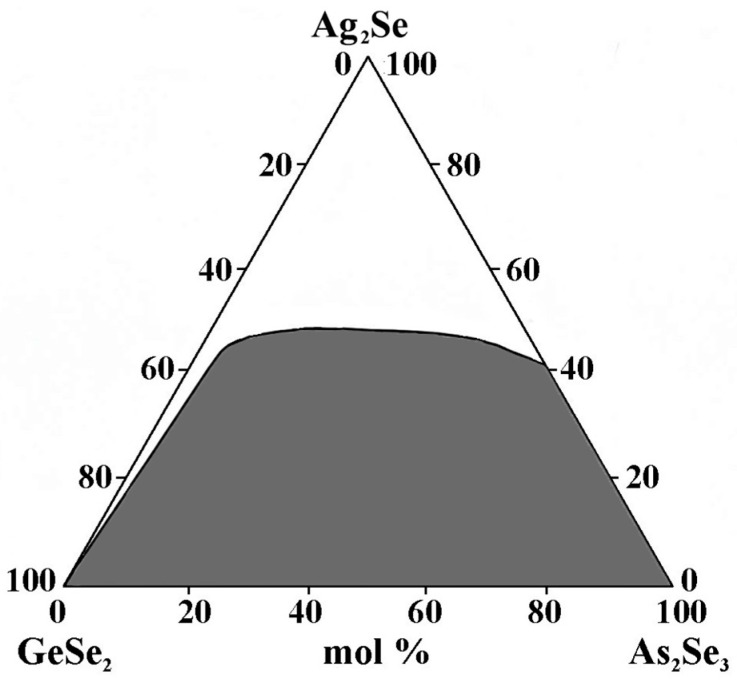
Glass formation in the GeSe_2_–As_2_Se_3_–Ag_2_Se system, adapted from Ref. [24].

The GeSe_2_–As_2_Se_3_–Ag_2_Se glass-forming region lies entirely on the GeSe_2_–As_2_Se_3_ side and incompletely on the As_2_Se_3_–Ag_2_Se side (Figure 2). No glasses are obtained on the GeSe_2_–Ag_2_Se side. Ag_2_Se exhibits a maximum solubility of 50 mol% GeSe_2_–As_2_Se_3_.

### 2.3. Glass Formation in the GeSe_2_–As_2_Se_3_–Ag_4_SSe System [26]

The compositional range over which glasses can form in the GeSe_2_–As_2_Se_3_–Ag_4_SSe system is delineated using 24 compositions of starting components As, Ge, Ag, S, and Se with a purity of 5N. GeSe_2_ and As_2_Se_3_ are synthesized using the regimes: GeSe_2_—the mixture is heated sequentially at 300, 620, and 950 °C for 2 h each, then cooled to 850 °C and quenched in an ice-water bath; As_2_Se_3_—the mixture is heated at 300 and 500 °C for 1 h each, followed by heating at 900 °C for 2 h, and then cooled in air. Ag_4_SSe is obtained by direct synthesis from Ag_2_S and Ag_2_Se; the maximal temperature is 1000 °C (the method for their preparation is described in [27]). The samples are directly monotemperature-synthesized in evacuated quartz ampules at temperatures 420 °C (1 h), followed by 900 °C (2 h) and 850 °C (0.5 h), and quenched with ice-water.

The synthesized samples are subjected to visual, XRD (TUR–M16, CuK_α_, Ni–filter), electron microscopic (Philips, Amsterdam, The Netherlands), and Raman analyses.

The obtained samples are observed visually using a binocular magnifying glass. The state is identified by the distinctive fracture of a recently exposed surface: glassy, crystalline, or glassy–crystalline (presence of two phases: crystalline + glassy). The glassy alloys from the studied systems exhibit a well-pronounced shell fracture, a metallic luster, and a dark color.

The diffraction patterns of the samples with compositions from the glass-forming region are typical of the amorphous state: an amorphous halo and the absence of diffraction reflexes. The X-ray patterns of the compositions that border the glass-forming region have a small number of clearly expressed diffraction reflexes of low intensity. Samples located outside the glass-forming region exhibit a diffraction pattern typical of the crystalline material—a large number of intense reflexes—indicating the presence of one or more phases. In the compositions rich in GeSe_2_, Se lines also appear due to the decomposition of GeSe_2_ to GeSe and Se. In the compositions rich in Ag_4_SSe, Ag_4_SSe lines are also recorded.

The electron microscopy reveals homogeneous, smooth surfaces for samples within the glass-forming region. Samples at the boundary of the region exhibit small crystalline areas on their surfaces, indicating a tendency towards crystallization in local regions of the glass. The samples falling outside the glass-forming region exhibit a rough, nonuniform surface, typical of a crystalline phase.

The Raman spectra of the most stable glasses containing 10 mol% Ag_4_SSe are investigated. Samples with the highest GeSe_2_ content exhibit a prominent band at 200 cm^−1^, accompanied by a shoulder near 215 cm^−1^, corresponding to heteropolar Ge–Se bond vibrations in GeSe_4_ tetrahedra, which dominate the glassy network. A broad feature centered around 320 cm^−1^ is attributed to Ge–Ge bonds within (Se_3_Ge–GeSe_3_) structural units. The incorporation of As_2_Se_3_ promotes the formation of AsSe_3/2_ pyramidal units, evidenced by the emergence of a distinct band at 230 cm^−1^ [26].

**Figure 3:**
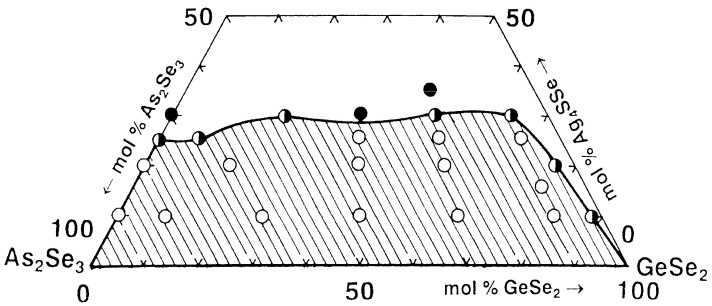
Glass formation in the GeSe_2_–As_2_Se_3_–Ag_4_SSe system. ○—glassy sample; ◑—glassy–crystalline sample; ●—crystalline sample. Reprinted with permission from Ref. [26]. Copyright 2005 Elsevier.

Based on the syntheses performed and the X-ray phase and electron microscopic analyses results, the glass formation in the GeSe_2_–As_2_Se_3_–Ag_4_SSe system is delineated (Figure 3). It lies entirely on the As_2_Se_3_–GeSe_2_ side and is partially located on the As_2_Se_3_–Ag_4_SSe side (from 0 to 25 mol% Ag_4_SSe). No glasses are obtained in the GeSe_2_–Ag_4_SSe system. The GeSe_2_–As_2_Se_3_ pair dissolves up to about 25 mol% Ag_4_SSe.

### 2.4. Glass Formation in the GeSe_2_–As_2_Se_3_–ZnSe System [28]

The samples are prepared, using a monothermal synthesis method, from high-purity elements: germanium, selenium, and tellurium with 5N purity, arsenic, and zinc selenide with 4N purity [28]. The synthesis is carried out in accordance with the physicochemical properties of the starting components and intermediate phases.

**Figure 4:**
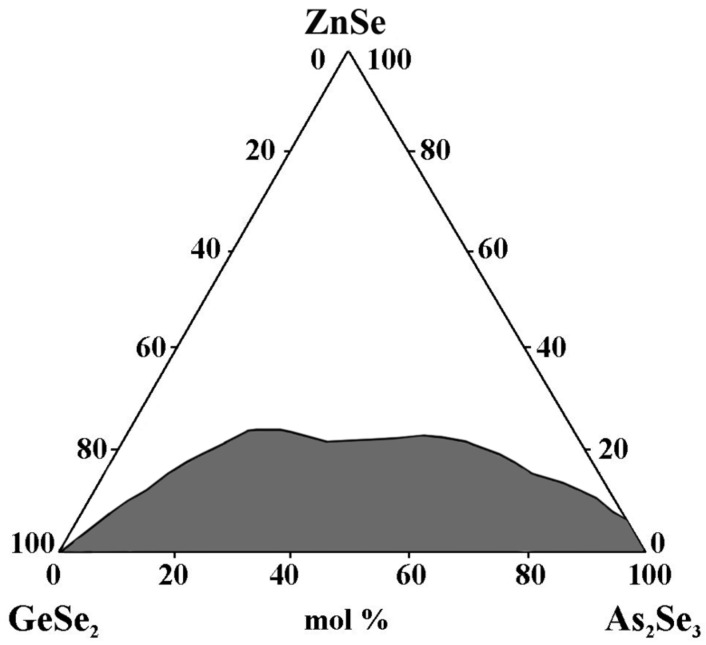
Glass formation in the GeSe_2_–As_2_Se_3_–ZnSe system, adapted from Ref. [28].

The glass-forming region lies entirely on the GeSe_2_–As_2_Se_3_ side and is located from 0 to 8 mol% ZnSe on the As_2_Se_3_–ZnSe side (0 to 8 mol% ZnSe) (Figure 4). The maximum solubility of ZnSe is 22 mol%. During the crystallization of the glasses, the ZnSe + GeSe_2_ and ZnSe + As_2_Te_3_ reflexes are registered [28].

### 2.5. Glass Formation in the GeSe_2_–As_2_Se_3_–ZnTe System [29]

To delineate the region of glass formation in the GeSe_2_–As_2_Se_3_–ZnTe system, 22 samples are synthesized from high-purity initial elements: As, Ge, and Se with a purity of 5N, as well as ZnTe with a purity of 4N (Balzers). The synthesis is carried out with three temperature holds at 420 °C (1 h), 800 °C (1 h), and 1150 °C (2 h), and vibrational agitation of the melt during the final stage. The samples are rapidly cooled in a water–ice mixture.

The state of the samples is determined using visual, microscopic, and X-ray phase analyses. A binocular magnifying lens is used to examine the samples. According to the characteristic fracture of a freshly exposed surface, the state of the samples is determined as glassy, crystalline, or glassy–crystalline. The glassy specimens are dark-colored with a strong luster. The crystalline samples have a matte, rough surface, whereas the presence of both phases distinguishes the glassy–crystalline samples located on the boundary.

Indexing of the diffractograms shows that no peaks of the initial compounds are observed. The ZnGeAs_2_ phase is registered in all samples. Glassy samples also exhibited peaks of zinc telluride, tellurium, selenium, germanium selenide, and arsenic selenide. In the course of high-temperature synthesis, GeSe_2_ decomposes to GeSe and Se; selenium links the GeSe_2_ structural units, forming continuous linear chains. In the As-rich glasses, amorphous AsSe–reflexes are observed.

Glassy samples have smooth, uniform surfaces with a few microcrystalline areas visible under a microscope, whereas samples beyond the glassy region are fully crystalline.

**Figure 5:**
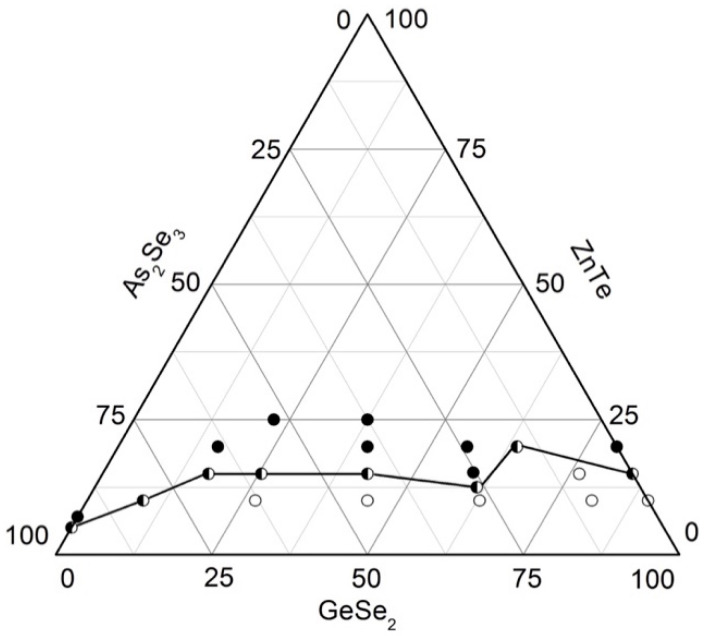
Glass formation in the GeSe_2_–As_2_Se_3_–ZnTe system [29]. ○—glassy sample; ◑—glassy–crystalline sample; ●—crystalline sample.

Based on the syntheses and analyses, the glass formation in the system As_2_Se_3_–GeSe_2_–ZnTe (Figure 5) is outlined. It lies entirely on the GeSe_2_–As_2_Se_3_ side, from 0 to 15 mol% ZnTe on the GeSe_2_–ZnTe and from 0 to 5 mol% As_2_Se_3_ on the As_2_Se_3_–ZnTe sides. The solubility of ZnTe in the glasses reaches 20 mol% [29].

### 2.6. Glass Formation in the GeSe_2_–As_2_Se_3_–CdSe System [30,31]

Two groups have studied the formation of GeSe_2_–As_2_Se_3_–CdSe glass. Different regions are identified, likely due to differences in the samples’ synthesis regimes. In both regions, a continuous series of glasses in the GeSe_2_–As_2_Se_3_ binary system has not been identified, as has been reported in the other systems considered.

Glass formation in the GeSe_2_–As_2_Se_3_–CdSe system was studied by Pazin et al. [30]. The alloys were synthesized directly from the corresponding elements at T_max_ = 950 °C. The melts were quenched in air from 850 °C.

**Figure 6:**
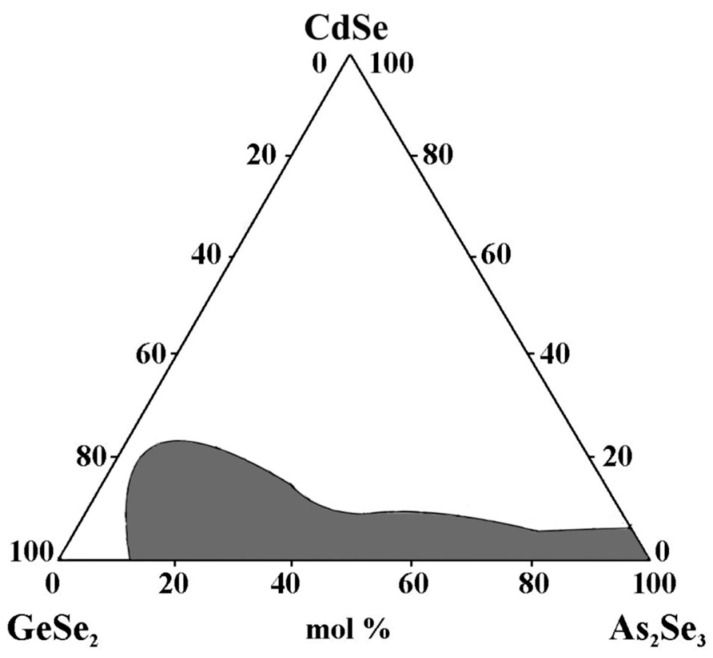
Glass formation in the GeSe_2_–As_2_Se_3_–CdSe system, adapted from Ref. [30].

An area of chalcogenide glasses is obtained (Figure 6), with the maximum solubility of CdSe 20 mol% (~6 at% Cd). The alloys near the eutectic in the GeSe_2_–As_2_Se_3_ section exhibit the highest glass-forming ability. In the CdSe–GeSe_2_ system, no glasses are obtained, while in the As_2_Se_3_–CdSe system, glasses containing CdSe < 5 mol% are obtained [30]. A vast area of chalcogenide glasses is formed along the GeSe_2_–As_2_Se_3_ section (Figure 6), whose matrix structural units or their small associates containing Cd are statistically distributed.

**Figure 7:**
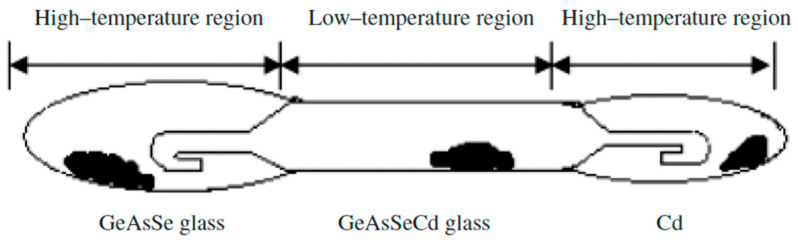
Ampoule design for the distillation process. Reprinted with permission from Ref. [31]. Copyright 2005 John Wiley and Sons.

Zhao Donghui et al. [31] also studied the glass formation in this system. High-purity elements (5N)—germanium, arsenic, selenium, and cadmium—are used to synthesize the samples. A distillation process is carried out to obtain high-purity glasses. The pre-synthesized glasses containing Ge, As, and Se are loaded into an evacuated ampoule with a specific design containing Cd. Figure 7 shows the distillation ampoule, where the pre-synthesized glass and Cd are located at both ends of the ampoule (high-temperature region). After distillation, Ge, As, Se, and Cd are evaporated to the low-temperature (middle) part; however, because of the low partial pressure at elevated temperatures, the oxides are still present. To completely react the components, the center section is then sealed and heated to 850 °C for 10 h in a swing furnace. The melt is cooled in air or water. The resulting glass is annealed at 300 °C for 3 h. The homogeneity and absence of crystallinity in the samples are verified using thermal imaging and X-ray diffraction analysis. XRD analysis reveals the formation of Cd_4_GeSe_6_, indicating Cd-rich clustering within the glass network. This localized structure reduces stability and promotes crystallization, confirming Cd’s role as a nucleating agent [31].

**Figure 8:**
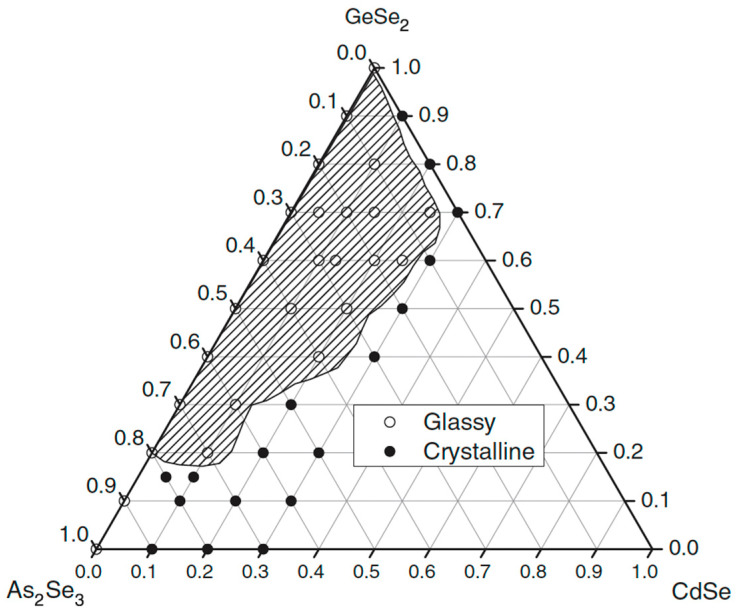
Glass formation in the GeSe_2_–As_2_Se_3_–CdSe system. Reprinted with permission from Ref. [31]. Copyright 2005 John Wiley and Sons.

Figure 8 presents the glass formation in the GeSe_2_–As_2_Se_3_–CdSe system [31]. A broad glassy area is observed, where the amount of added Cd can reach 8.77 at%. Glass formation mainly occurs along the GeSe_2_–As_2_Se_3_ side, consistent with the strong glass-forming ability of the structural units [GeSe_4_] and [AsSe_3_]. The GeSe_2_-rich compositions accommodate more Cd compared to the As_2_Se_3_-rich region, although the overall tendency toward crystallization increases as the Cd content rises. Cadmium atom solubility is enhanced by the presence of [GeSe_4_] units. The XRD data indicate that cadmium atoms alter the glass network and are anticipated to act as nucleating agents in the microcrystallization process. This experimental finding implies that CdSe can participate more fully in this glass system due to the increased GeSe_2_ concentration. No glass with a composition along the GeSe_2_–CdSe line is produced in this study.

### 2.7. Glass Formation in the GeSe_2_–As_2_Se_3_–CdTe System [32]

Twenty-four compositions are used to identify the glass formation in the GeSe_2_–As_2_Se_3_–CdTe system. High-purity initial materials are employed: Ge, As, Se (5N), and CdTe (BALZERS, 99.999% purity). Depending on the CdTe concentration, the precursors GeSe_2_ and As_2_Se_3_, as well as the system samples, are directly synthesized at 850–1000 °C. The melts are held at the maximum synthesis temperature for approximately 2 h with vibrational stirring, then quenched in a water–ice mixture [32].

The synthesized samples of the GeSe_2_–As_2_Se_3_–CdTe system exhibit a dark color and a strong luster. XRD results show that, for compositions from the glass-forming region, no peaks are observed in the diffractograms. Low-intensity diffraction peaks are presented in other compositions (glassy–crystalline samples at the boundary). In contrast, other diffractograms are characterized by well-defined peaks (crystalline samples lying outside the glass-forming region). On the XRD patterns of the glassy–crystalline and crystalline samples, the reflections of the compounds GeSe_2_ and As_2_Se_3_ are observed. In GeSe_2_-rich alloys, another line is registered, identified as the strongest Se line, obtained from the partial decomposition of GeSe_2_ [32].

The surfaces of the glassy samples are smooth and uniform when examined under an electron microscope. The samples outside the glass-forming region are completely crystalline, while those at the border have small crystalline regions visible on their surfaces.

The glassy samples’ surfaces are homogeneous and smooth under an electron microscope. Tiny crystalline areas are noticeable on the surface of the samples that border the glass-forming region, while the samples lying outside the glass area are entirely crystalline.

**Figure 9:**
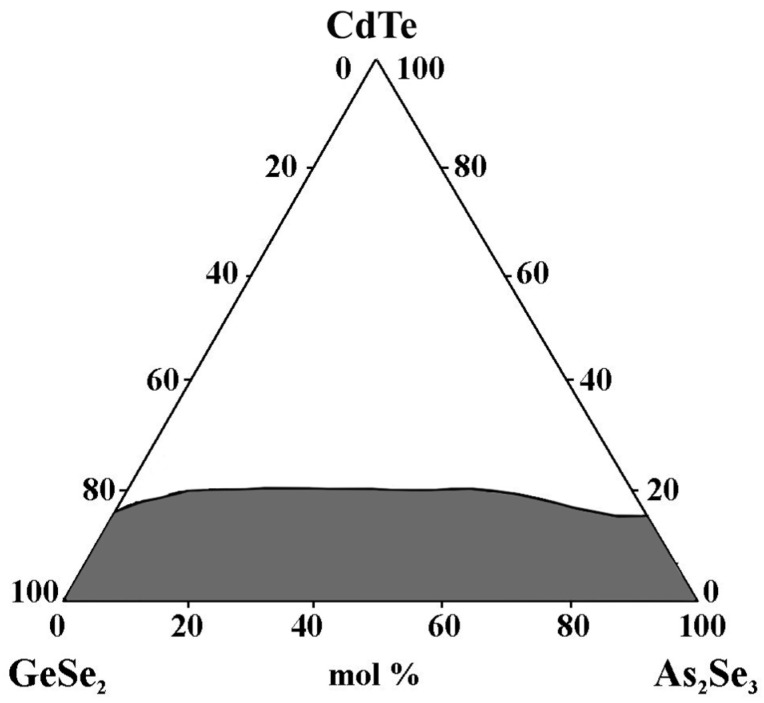
Glass formation in the GeSe_2_–As_2_Se_3_–CdTe system, adapted from Ref. [32].

Visual inspection, XRD, and electron microscopy are used to identify the glass-forming area in the GeSe_2_–As_2_Se_3_–CdTe system. It is entirely on the GeSe_2_–As_2_Se_3_ side and from 0 to 17 mol% CdTe on the GeSe_2_–CdTe and As_2_Se_3_–CdTe sides (Figure 9). The maximum solubility of CdTe reaches up to 20 mol% CdTe [32].

### 2.8. Glass Formation in the GeSe_2_–As_2_Se_3_–PbSe System [33]

The alloys from the GeSe_2_–As_2_Se_3_–PbSe system are prepared using a conventional melt-cooling method. High-purity elements Ge, As, Se, and Pb (5N) are refined to remove impurities such as molecular H_2_O, O_2_, C, Si, and heavy metals. All components in the designated amounts are put into specific-shaped ampoules [31], evacuated (10^3^ Pa), and sealed. The sealed ampoules are heated to 850 °C in a vibrating furnace for 10 h to ensure complete reaction, then water-quenched.

**Figure 10:**
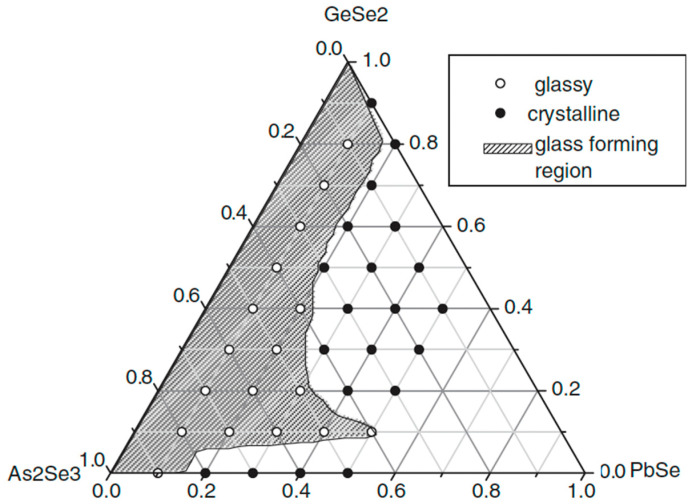
Glass formation in the GeSe_2_–As_2_Se_3_–PbSe system. Reprinted with permission from Ref. [33]. Copyright 2007 John Wiley and Sons.

The glass formation in the GeSe_2_–As_2_Se_3_–PbSe system is presented in Figure 10 [33]. With a maximum of 15.15 at% Pb added, the system has relatively good glass-forming ability.

GeSe_2_ and As_2_Se_3_ are good glass formers, which is consistent with the fact that glasses can form throughout the entire GeSe_2_–As_2_Se_3_ phase. Furthermore, the As_2_Se_3_-rich area could incorporate a greater Pb content than the GeSe_2_-rich region.

### 2.9. Glass Formation in the GeSe_2_–As_2_Se_3_–SnSe_2_ System [34]

The alloys are obtained by alloying elemental substances (As, Se, Ge, and Sn) with a high degree of purity in quartz ampoules evacuated to 10^−3^ mm Hg. The synthesis is performed at 950 °C for 4 h with continuous vibratory stirring. Quenching is carried out in the air [34]. In the GeSe_2_–As_2_Se_3_–SnSe_2_ system, 32 alloys from the concentration triangle (through 10 mol%) are synthesized. As criteria for the glassy state, X-ray amorphousness and uniformity, when observing a polished surface with a metallographic microscope, are used.

**Figure 11:**
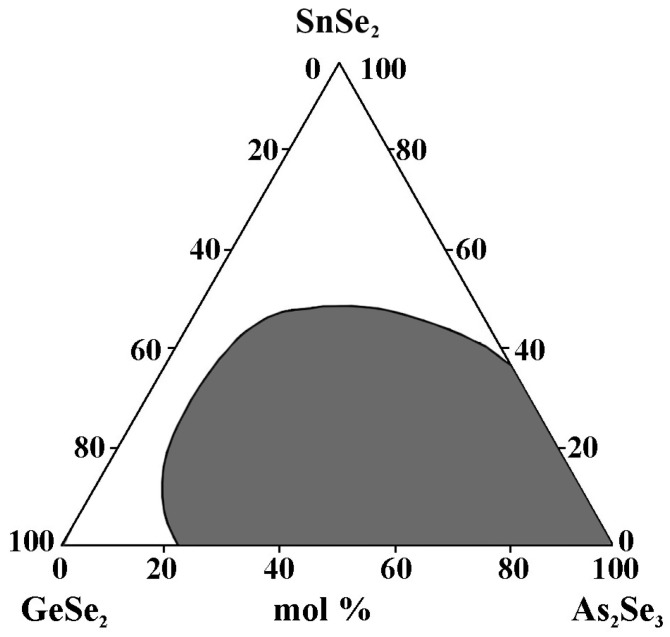
Glass formation in the GeSe_2_–As_2_Se_3_–SnSe_2_ system, adapted from Ref. [34].

The glass formation region (Figure 11) is located on the GeSe_2_–As_2_Se_3_ (22–100 mol% As_2_Se_3_) and As_2_Se_3_–SnSe_2_ (0–35 mol% SnSe_2_) sides.

### 2.10. Glass Formation in the GeSe_2_–As_2_Se_3_–SnTe System [26]

The glass formation in the GeSe_2_–As_2_Se_3_–SnTe system is delineated using 30 compositions synthesized via direct monotemperature synthesis from the elements arsenic, germanium, selenium, tellurium (5N), and tin (3N5). The samples are synthesized at a maximal temperature of 900 °C for 2 h and quenched in a water–ice mixture [26].

Visual analysis of the samples revealed that the glassy alloys exhibit a well-defined shell-like fracture, metallic luster, and dark color. The SnTe lines are registered on the XRD patterns of the SnTe-rich samples. Electron microscopy analysis reveals that the samples located in the glass regions have a homogeneous and smooth surface. Small crystalline regions are observed on the surfaces of the samples located along the boundaries of the glass transition regions, i.e., the tendency towards crystallization in local areas of the total glassy mass is pronounced. Similar Raman features to those observed in the GeSe_2_–As_2_Se_3_–Ag_4_SSe system are observed in the SnTe-containing glasses, indicating comparable structural arrangements. This suggests that SnTe plays a role similar to that of Ag_4_SSe in stabilizing the glass network by forming Ge–Se and As–Se structural units [26].

**Figure 12:**
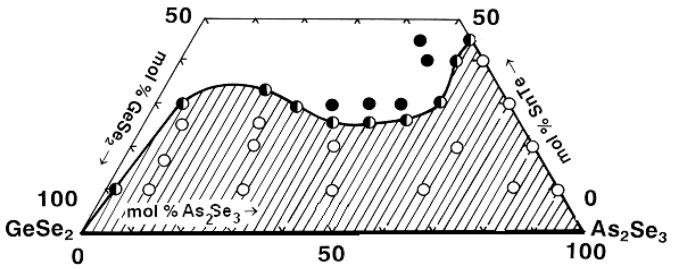
Glass formation in the GeSe_2_–As_2_Se_3_–SnTe system. ○—glassy sample; ◑—glassy–crystalline sample; ●—crystalline sample. Reprinted with permission from Ref. [26]. Copyright 2005 Elsevier.

Based on the syntheses and analyses conducted, the glass-forming area in GeSe_2_–As_2_Se_3_–SnTe (Figure 12) [26] is outlined. It lies on the GeSe_2_–As_2_Se_3_ side and from 0 to 45 mol% SnTe on the As_2_Se_3_–SnTe side. No glasses are obtained in the GeSe_2_–SnTe system.

## 3. Discussion

A characteristic feature of chalcogenide glasses, as well as glasses of other classes, is the increase in the tendency to form glass with an increase in the number of components in the alloy. The greater the complexity of an alloy’s composition, the more diverse the spatial arrangement of its structural units, which in turn makes the crystallization of specific phases more difficult [35,36]. Among the physicochemical factors determining glass formation in chalcogenide systems, Dembowski lists the presence of a glass-forming chemical compound first. According to various theories explaining the causes of glass formation (structural–chemical, kinetic, thermodynamic), two main factors of its formation can be distinguished: structural–chemical, which takes into account the mutual arrangement of atoms and the strength of the chemical bond, and kinetic–thermodynamic, or energy, which can be measured by the liquidus (melting) temperature [36].

Various scientific groups study the glass formation in several systems and the influence of changing one of the initial components. For glass formation in binary and ternary systems, objective regularities have been established about the area of the glass formation and the ordinal number of the elements, as well as the presence of a metallic chemical bond on the other hand. Various cases of inversion in the glass-forming ability of binary and ternary systems have also been established, which significantly complicates the identification of similar dependencies in multicomponent systems.

The addition of metallic elements (Me = Cu, Ag, Zn, Cd, Sn, Pb) to the GeSe_2_–As_2_Se_3_–Ch systems significantly influences the structural network and electronic structure of the glasses, affecting the glass-forming ability, stability, mechanical, optical, and electrical properties [37,38]. The metals act as network modifiers, partially replacing Ge–Se or As–Se bonds with Me–Ch bonds, thereby altering the average coordination number and the cross-linking density of the glass network.

When finding objective regularities in the change in the glass-forming areas in systems of the type GeSe_2_–As_2_Se_3_–MeCh, where Me = Cu, Ag, Zn, Cd, Sn, Pb; Ch = Se, Te, the following should be borne in mind:

- The systems are studied at different times, using different methods to prove the glassy state, and different goals are pursued; i.e., the studies conducted are not subordinated to a single idea.
- In the synthesis of the glasses, different ampoules are used in terms of volume and various amounts of charge, and the cooling of the melt is carried out at different speeds.

**Figure 13:**
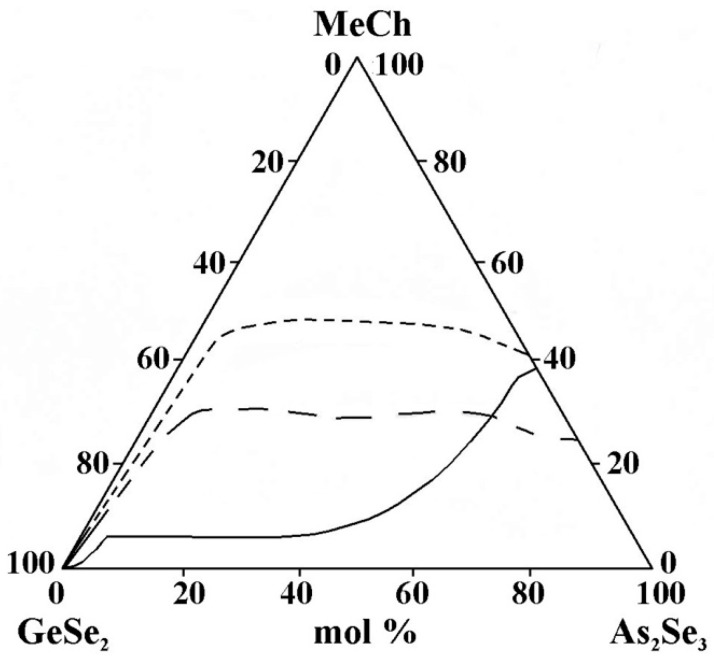
Glass formation in the GeSe_2_–As_2_Se_3_–MeCh systems: line MeCh = Cu_2_Se; short dash MeCh = Ag_2_Se; long dash MeCh = Ag_4_SSe, adapted from Refs. [21,24,26].

**Figure 14:**
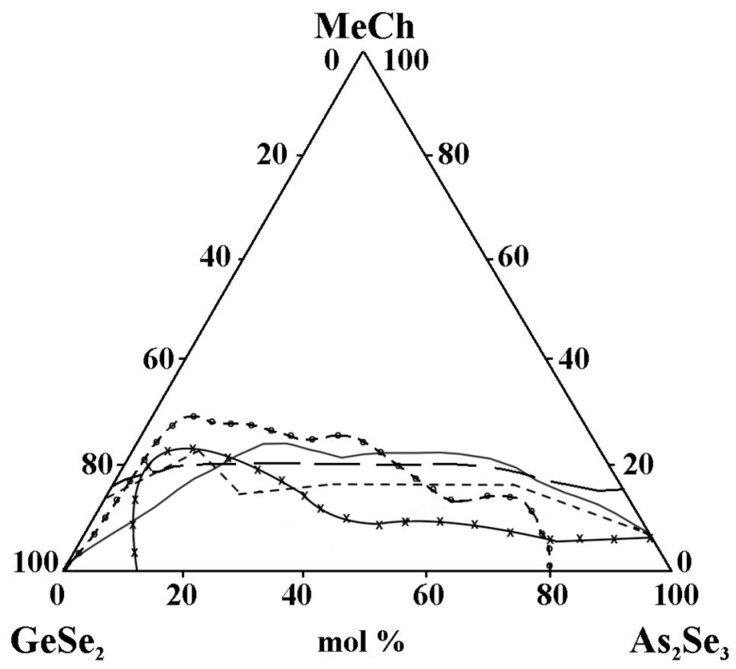
Glass formation in the GeSe_2_–As_2_Se_3_–MeCh systems: line MeCh = ZnSe; short dash MeCh = ZnTe; –x– MeCh = CdSe; –o– MeCh = CdSe; long dash MeCh = CdTe, adapted from Refs. [28,29,30,31,32].

**Figure 15:**
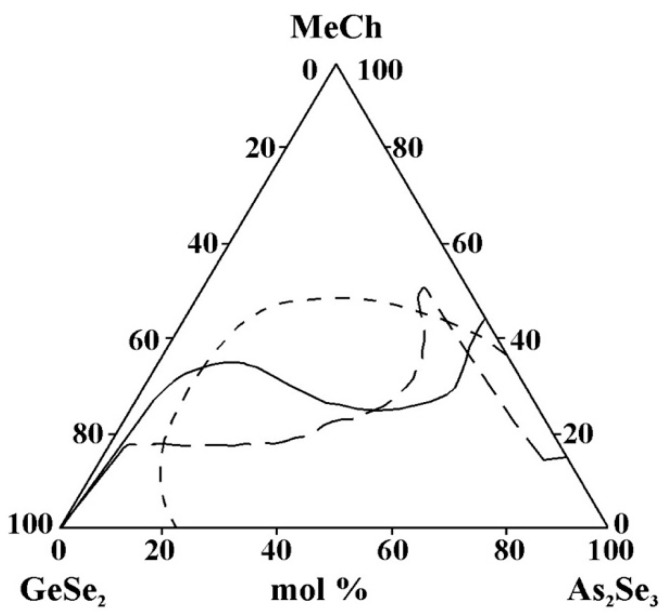
Glass formation in the GeSe_2_–As_2_Se_3_–MeCh systems: line MeCh = SnTe; short dash MeCh = SnSe_2_; long dash MeCh = PbSe, adapted from Refs. [26,33,34].

However, when comparing the considered areas of glass formation, the following regularities are observed:

- In the binary systems MeCh–As_2_Se_3_ (Me = Cu, Ag, Zn, Cd, Sn, Pb; Ch = Se, Te), a different amount of the metal chalcogenides MeCh is dissolved (8 mol% ZnSe to 45 mol% SnTe), while in the binary system GeSe_2_–MeCh, only the tellurides of cadmium and zinc are dissolved (up to 17 mol% CdTe and up to 15 mol% ZnTe).
- Studies of the glass formation in the systems GeSe_2_–As_2_Se_3_–MeCh [21,24,26,28,29,33,34] show that in the binary system GeSe_2_–As_2_Se_3,_ a continuous series of chalcogenide glasses is formed in the entire concentration range (from 0 to 100 mol% GeSe_2_), which aligns with the known glass-forming ability of GeSe_2_ and As_2_Se_3_. The only exception to this pattern is the GeSe_2_–As_2_Se_3_–CdSe system [30,31]; both research groups do not obtain glassy samples over 0 to 100 mol% As_2_Se_3_.
- The replacement of Cu by Ag in the metal selenide significantly expands the glass-forming area (Figure 13). The observed trend provides evidence of an inversion in the glass-forming ability associated with changes in the ionic character of the chemical bonds in the compounds introduced into the glass-forming matrix. Compounds and alloys with a predominantly covalent bonding character exhibit an enhanced tendency to form glasses in chalcogenide systems. In the order Cu_2_Se→Ag_2_Se, the ionic bonding character increases, which should hinder glass formation. The Cu–Se, Ag–Se, and Ag–S systems are non-glass-forming, as they exhibit melt phase separation and a significant increase in the liquidus temperature even with small additions of the second element to the chalcogen. Adding another chalcogenide (in this case, sulfur) reduces the region.
- The replacement of ZnTe with ZnSe slightly improves the glass-forming ability of the GeSe_2_–As_2_Se_3_ pair, expressed in the expansion of the geometric dimensions of the glassy region (Figure 14). This trend is confirmed by the rule that chalcogenide compounds with mostly covalent bonds have a greater propensity to form glass. In the order ZnSe → ZnTe, the ionic bonding character increases and hinders glass formation. In the GeSe_2_–As_2_Se_3_–CdSe system, studies conducted by two independent research groups report differing glass-forming regions, thereby complicating a consistent interpretation of the compositional dependence. According to the reported glass-forming regions [30,31,32], substitution of CdTe with CdSe increases the maximum solubility of the metal chalcogenide in the GeSe_2_–As_2_Se_3_ system (Figure 14); however, the replacement does not lead to a clear glass-formation trend. A deviation from these regularities for binary systems is described by Minaev [36]: the glass-forming ability of elements of group VIA first increases with atomic number up to selenium, then decreases oxygen < sulfur < selenium > tellurium > polonium. Thus, there is an inversion (reversal, rearrangement) in the basic regularity of glass formation, which should decrease with increasing atomic mass. Still, it increases during the transition from oxygen to sulfur and from sulfur to selenium. The replacement of CdTe with ZnTe in the GeSe_2_–As_2_Se_3_–CdTe system (Figure 14) slightly decreases the glass-forming ability of the GeSe_2_–As_2_Se_3_ pair. The observed trend is confirmed by the regularity according to which, when replacing an atom with one with a smaller atomic radius, rCd(48) > rZn(30), the region of glass formation expands. The replacement of CdSe with ZnSe in the GeSe_2_–As_2_Se_3_–CdSe system, described by Pazin et al. in [30], is a deviation from the above regularity. The glass-forming region, as it is described by Zhao et al. in [31], does not change significantly when replacing CdSe with ZnSe. When replacing CdSe with ZnSe, a different maximal solubility of CdSe/ZnSe in the GeSe_2_–As_2_Se_3_ is also observed (Figure 14).
- For systems containing Sn and Pb, data are found only on glass formation in systems with a third component, SnTe, SnSe_2_, and PbSe. A direct comparison of glass formation in the GeSe_2_–As_2_Se_3_ systems modified with SnTe, SnSe_2_, and PbSe is not straightforward, as the substitution alters both the metallic and chalcogenide constituents. Moreover, replacing Sn^4+^ with Pb^2+^ alters the oxidation state, thereby influencing the glass-forming ability. Nevertheless, as illustrated in Figure 15, the glass-forming region is observed to narrow upon substitution of Sn^4+^ by Pb^2+^. This observation appears to contradict the report in [36], which states that divalent lead, unlike Sn^+2^ and Sn^+4^, has a much greater ability to integrate into chalcogen chains and form bridging bonds with other elements, thereby enhancing the glass-forming ability. This discrepancy may be linked to the well-established Pb ↔ Sn inversion that occurs in binary systems, as well as in the quaternary systems Sn(Pb)–Ge–As–Se [36]—glassy alloys of these systems can contain 20 at% lead and only 15 at% tin. This inversion is expected to also occur in multicomponent alloys with tellurium (particularly in ternary alloys), as well as in alloys with lighter chalcogens, since it is not the structural features of the competition diagrams that are determined, but the difference in the chemical nature of lead and tin [36].

The inversions in the glass-forming ability described in [36] are valid for binary systems. For the considered multicomponent systems, the experimentally established inversion in period 5 ⟷ 4 of the Te ⟷ Se type and the inversion in period 6 ⟷ 5 of the Pb ⟷ Sn type, as well as the predicted inversion of the Ag ⟷ Cu type, have been confirmed. The comparison of the glass formation in the GeSe_2_–As_2_Se_3_–MeCh systems, where Me = Zn, Cd; Ch = Se, Te, establishes the presence of an inversion in period 5 ⟷ 4 of the Cd ⟷ Zn type.

## 4. Conclusions

The information regarding glass formation in the chalcogenide systems GeSe_2_–As_2_Se_3_–MeCh, where Me = Cu, Ag, Zn, Cd, Sn, Pb; Ch = S, Se, Te, was subjected to a thorough comparative analysis to establish objective regularities in the change in the glass-forming ability in the described systems. The influence of MeCh on the formation of glass in the binary system As_2_Se_3_–GeSe_2_ was traced. For the considered multicomponent systems, period 5 ⟷ period 4 inversions of the Te ⟷ Se type and Cd ⟷ Zn type have been confirmed, as well as period 6 ⟷ period 5 inversions of the Pb ⟷ Sn type and of the Ag ⟷ Cu type.

The next step after defining the glass-forming areas is a thorough examination of the physicochemical, electrical, and optical properties of the glassy samples. Since the reported glass-forming regions have been established by different research groups using various synthesis regimes, and different properties have been investigated, a direct comparison of results is challenging. Correlations between composition and properties, particularly the influence of metallic chalcogenide additions, remain unclear. Therefore, future research should focus on synthesizing glassy samples from these systems, altering only the metallic chalcogenide component, to establish clear correlations among structure, composition, and functional characteristics and identify their potential practical applications.

## Data Availability

No new data were created or analyzed in this study. Data sharing is not applicable to this article.
